# Fermented Rice Bran Supplementation Inhibits LPS-Induced Osteoclast Formation and Bone Resorption in Mice

**DOI:** 10.3390/nu15133044

**Published:** 2023-07-05

**Authors:** Takahiro Noguchi, Hideki Kitaura, Aseel Marahleh, Afifah Zahra Agista, Yusuke Ohsaki, Hitoshi Shirakawa, Itaru Mizoguchi

**Affiliations:** 1 Division of Orthodontics and Dentofacial Orthopedics, Tohoku University Graduate School of Dentistry, 4-1, Seiryo-machi, Aoba-ku, Sendai 980-8575, Miyagi, Japan; takahiro.noguchi.d4@tohoku.ac.jp (T.N.); itaru.mizoguchi.c3@tohoku.ac.jp (I.M.); 2 Frontier Research Institute for Interdisciplinary Sciences (FRIS), Tohoku University, 6-3, Aramaki-Aoba, Aoba-ku, Sendai 980-0845, Miyagi, Japan; aseel.mahmoud.suleiman.marahleh.e6@tohoku.ac.jp; 3 Laboratory of Nutrition, Graduate School of Agricultural Science, Tohoku University, 468-1, Aramaki Aza Aoba, Aoba-ku, Sendai 980-8572, Miyagi, Japan; agista@g-mail.tohoku-university.jp (A.Z.A.); yusuke.ohsaki.a4@tohoku.ac.jp (Y.O.); shirakah@tohoku.ac.jp (H.S.)

**Keywords:** osteoclast, bone resorption, fermented rice bran, LPS, TNF-α, RANKL

## Abstract

Fermented rice bran (FRB) is known to have numerous beneficial bioactivities, amongst which is its anti-inflammatory properties when used as a supplement. To determine its effects, we examined osteoclastogenesis and bone resorption caused by injections of lipopolysaccharide (LPS), using mice with and without FRB supplementation. The results were favorable: those that received FRB showed reduced osteoclast numbers and bone resorption compared to those with the control diet. Notably, receptor activator of NF-κB ligand (RANKL) and tumor necrosis factor-α (TNF-α) mRNA levels were shown to be lower in the LPS-treated animals with FRB supplementation. FRB’s inhibitory effect on RANKL- and TNF-α-induced osteoclastogenesis was further confirmed in vitro. In culture, macrophages exhibited decreased TNF-α mRNA levels when treated with FRB extract and LPS versus treatment with LPS alone, but there was no significant change in RANKL levels in osteoblasts. We can conclude that FRB supplementation dampens the effect of LPS-induced osteoclastogenesis and bone resorption by controlling TNF-α expression in macrophages and the direct inhibition of osteoclast formation.

## 1. Introduction

Osteoclasts are multinucleated cells that have the ability of bone resorption. Osteoclasts differentiate via the fusion of osteoclast precursors originating from hematopoietic bone marrow cells and usually exist on the bone surface [1]. Osteolysis, caused by osteoclasts, is a key complication in diseases such as aseptic loosening, periodontal disease, and rheumatoid arthritis [2,3,4,5]. Diseases characterized by pathological bone resorption and inflammation have a detrimental impact on everyday activities and significantly diminish one’s quality of life. Therefore, it is imperative to understand the underlying mechanisms of such conditions and discover methods to treat them effectively. Several cytokines are known to affect osteoclast formation and bone resorption. Macrophage colony stimulating factor (M-CSF) and receptor activator of NF-κB ligand (RANKL) are obligatory cytokines for osteoclast differentiation [4,5]. Tumor necrosis factor (TNF)-α is also a cytokine known to directly induce osteoclast formation in vitro [6,7] and in vivo [8,9].

Lipopolysaccharide (LPS) is found abundantly in natural environments like soil, fields, and forests. It constitutes a significant part of the outer membrane of Gram-negative bacteria. It is composed of polysaccharides and lipid A. LPS is widely recognized for its ability to trigger inflammation and stimulate the production of inflammatory cytokines contributing to pathological bone loss [10]. By inducing the production of a cocktail of cytokines, LPS impacts the differentiation and maturation of osteoclasts [11,12]. LPS affects osteoclast formation and bone resorption in vitro and in vivo [13] and enhances osteoclast survival and fusion [14]. LPS has also been shown to upregulate RANKL expression in osteoblasts [15], and it is recognized as a major causal factor of osteolytic inflammatory diseases such as periodontitis, osteomyelitis, septic arthritis, and implant infections [16]. Hence, identifying ways to inhibit LPS-induced pathological bone resorption can mitigate the disadvantages associated with these diseases.

The bran fraction of rice is considered an agricultural waste; however, rice bran (RB) has recently gained attention for its high nutritional value. RB includes bioactive components with various functional properties [17,18,19]. The components have been shown to have a potential role in disease prevention and treatment [17,20,21,22]. It has also been reported that rice bran has anti-inflammatory effects [23,24]. Despite its benefits, the consumption of RB by humans has been limited because of its instability, making it challenging to blend RB into a variety of foods. RB is prone to decomposition when not treated, hindering its development as a food ingredient or supplement [17,25,26]. Several studies have shown that a double fermentation process can be implemented to make RB more appropriate as a food material or supplement. Fermented rice bran (FRB) was produced via double fermentation with *Aspergillus kawachii* and three lactic acid bacteria. *Aspergillus kawachii* is a common bacterium used in the fermentation process of Japanese shochu [27,28]. *Aspergillus kawachii* produces a variety of hydrolytic enzymes during the fermentation process, including amylase, protease, and lipase. These enzymes break down the complex molecules in rice into smaller molecules. The small molecules are used by other microorganisms such as Bacillus subtilis and Saccharomyces cerevisiae [27,28]. Double fermentation means that the fermentation with *Aspergillus kawachii* is further fermented with lactic acid bacteria. Lactic acid bacteria, such as *Enterococcus faecium*, *Levilactobacillus brevis*, and *Lacticaseibacillus rhamnosus*, can be found in various fermented foods which are commonly known to act as probiotics [29,30,31,32]. This combination of microorganisms is anticipated to increase the nutritional value of RB. It has been shown in previous studies that this FRB can protect the intestine of mice from dextran sodium sulfate (DDS)-induced acute colitis [33], extraintestinal manifestation [34] and the progress of intestinal fibrosis caused by DDS-induced inflammation in mice [35]. FRB can reduce the expression of markers of inflammation in the muscles of diabetic rats [36]. Moreover, FRB suppresses metabolic syndromes in stroke-prone spontaneously hypertensive rats [37]. It has been reported that this double fermentation process increased RB’s nutritional qualities, especially the total phenolic content [37]. Double fermentation increases the microbial tryptophan metabolites and the concentration of tryptophan in RB [33]. In recent years, it has been reported that tryptamine, one of FRB’s components, inhibited LPS-induced inflammation in a mouse macrophage model [38]. This change in nutritional status may contribute to the anti-inflammatory effects of FRB. In this way, FRB has the effect of suppressing inflammation. We hypothesized that FRB could inhibit diseases associated with pathological bone resorption caused by inflammation.

The purpose of this study was to evaluate the effects of FRB (made from a double fermentation of *Aspergillus kawachii* and three lactic acid bacteria) on osteoclast formation and bone resorption upon LPS injections in an inflammatory animal model and clarify the mechanisms by experimental in vitro studies.

## 2. Materials and Methods

### 2.1. Material and Reagents

Briefly, FRB was prepared via a two-step fermentation with yeast mold and lactic acid bacteria. First, rice bran was fermented with *Aspergillus kawachii* at 30 °C for 44 h, then mixed with rice flour in a 2:1 ratio. The mixture was saccharified by adding four times as much water and incubating at 56 °C for 12 h. The saccharified solution obtained in this stage was treated at 85 °C for 15 min and then cooled to about 30 °C. In the second step, three lactic acid bacteria (*Enterococcus faecium*, *Lactobacillus rhamnosus*, and *Lactobacillus brevi*) were added (0.01% (*w*/*w*)) and fermented overnight at 37 °C. After each fermentation, the FRB solution was further heated at 85 °C for 15 min, filtered, and lyophilized. The lyophilized powder was used as an FRB diet. In vitro experiments, the supernatant obtained by dissolving lyophilized FRB in dimethyl sulfoxide (DMSO) was used as FRB extract. Recombinant mouse RANKL was obtained from PeproTech (Rocky Hill, NJ, USA), recombinant mouse TNF-α was obtained from R&D Systems (Minneapolis, MN, USA), and LPS from *Escherichia coli* was obtained from Sigma-Aldrich (St. Louis, MO, USA). In addition, murine M-CSF was taken from CMG14-12 cells, which is an M-CSF expressing cell line, as described previously [39]. 

### 2.2. Animals

Male 8–10-week-old mice (C57BL6/J) were purchased from CLEA Japan (Tokyo, Japan). All mice were housed in individual pathogen-free gauges under a 12 h light-dark cycle at Institute for Animal Experimentation, Tohoku University. All experimental procedures were performed in accordance with the Guidelines for Animal Experiments of Tohoku University. The Animal Care Committee of Tohoku University approved the experimental plan of this study. In this study, a control diet (AIN93M standard diet) and 10% FRB based on the AIN93M diet were introduced 7 days before and during the injection of reagents. Diet and water were introduced ad libitum. 

### 2.3. Preparation for Histological Evaluation

Following the protocol of our previous study [40], subcutaneous injections of LPS in the mouse calvaria were performed to activate osteoclastogenesis by inducing local inflammation. Mice were divided into four groups and injected subcutaneously with phosphate-buffered saline (PBS) or LPS (100 μg/day) for 5 days under the feeding of each diet, and each mouse was sacrificed on day 6. To obtain paraffin sections, the calvaria were immediately excised after sacrifice, fixed with 4% formaldehyde, and then decalcified with 14% ethylenediaminetetraacetic acid (EDTA). After softening the calvaria, they were cut into three pieces perpendicular to the sagittal midline, processed by an automated tissue processor (TP1020, Leica, Wetzlar, Germany), and embedded in paraffin. Using a microtome, samples were serially paraffin sectioned (5 µm). To visualize osteoclasts, TRAP staining was performed on the paraffin sections. TRAP stain solution was prepared as previously described [41]. Counterstaining was performed using hematoxylin (Wako, Osaka, Japan). TRAP-positive cells with three or more nuclei were considered active osteoclasts and were counted on five slides per section of the sagittal suture and then averaged [41].

### 2.4. Analysis of Bone Resorption Area by Micro-CT

Mouse calvariae collected after sacrifice were scanned using a microfocus CT (ScanXmate-E090, Comscan, Kanagawa, Japan) to evaluate the degree of inflammatory bone loss on the calvariae before tissue sections were prepared. After constructing three-dimensional images of mouse calvariae using image analysis software (TRI/3D-BON64; RATOC System Engineering, Tokyo, Japan), the percentage of bone resorption area to total area within a defined frame in the center of the calvaria was analyzed by Image J (NIH, Bethesda, MD, USA) [41].

### 2.5. Osteoclast Culture

Bone marrow cells were obtained from the isolated femur and tibia of male 8–10-week-old mice. The cells were cultured in α-MEM (Sigma Aldrich, St. Louis, MO, USA) supplemented with 10% fetal bovine serum (FBS), 1% antibiotics (penicillin, streptomycin) (Meiji Seika, Tokyo, Japan) and M-CSF (100 ng/mL). Three days later, adherent cells were harvested using trypsin-EDTA solution (Sigma Aldrich) and defined as osteoclast precursors. Harvested cells were seeded in a 96-well plate (5 × 10^4^ cells/well) and cultured in medium containing M-CSF (100 ng/mL) with 0.5% DMSO or FRB extract (500 μg/mL), M-CSF (100 ng/mL) and RANKL (100 ng/mL) or TNF-α (100 ng/mL) with 0.5% DMSO or different concentrations of FRB extract (10, 50, 100 and 500 μg/mL). DMSO was used as a control vehicle. After 5 days, cultured cells were fixed with 10% formalin solution, permeabilized with 0.2% Triton X-100, and stained by TRAP. TRAP-positive multinucleated cells (≥3 nuclei) were counted after imaging under an optical microscope [41]. Osteoclast precursors were cultured on Osteo Assay Plate 96 Well (Corning Life Sciences, Corning, NY, USA) for resorption pits assay. After 5 days, pit formation was evaluated [41]. Data were expressed as resorption pits area per total surface area.

### 2.6. Analysis of Expression Level Using Real-Time RT-PCR

Calvaria collected from mice were immersed in TRIzol reagent (Invitrogen, Carlsbad, CA, USA), crushed and centrifuged. Total RNA was purified using the RNeasy mini kit (Qiagen, Valencia, Santa Clarita, CA, USA) for in vivo experiments. For the in vitro experiments, both macrophages and osteoblasts were treated with PBS and 0.5% DMSO, LPS (100 ng/mL) and 0.5% DMSO, LPS (100 ng/mL) and FRB extract (500 μg/mL) or PBS and FRB extract (500 μg/mL) for 3 days; then, total RNA was extracted from their samples using the RNeasy mini kit (Qiagen). cDNA was synthesized from total RNA using Superscript IV reverse transcriptase (Invitrogen). Real-time RT-PCR was performed on the Thermal Cycler Dice Real-Time system (Takara, Shiga, Japan) using TB Green Premix Ex Taq II (Takara Bio). The specific primers used in this study were as follows: GAPDH (forward: 5′-GGTGGAGCCAAAAGGGTCA-3′, reverse: 5′-GGGGGCTAAGCAGTTGGT-3′); TRAP (forward: 5′-AACTTGCGACCATTGTTA-3′, reverse: 5′-GGGGACCTTTCGTTGATGT-3′); Cathepsin K (forward: 5′-GCAGAGGTGTGTACTATGA-3′, reverse: 5′-GCAGGCGTTGTTCTTATT-3′); TNF-α (forward: 5′-CTGTAGCCCACGTCGTAGC-3′, reverse: 5′-TTGAGATCCATGCCGTTG-3′); and RANKL (forward: 5′-CCTGAGGCCCAGCCATTT-3′, reverse: 5′-CTTGGCCCAGCCTCGAT-3′). All reactions were normalized with GAPDH as a reference gene [42].

### 2.7. Cell Viability Assay

It is possible that FRB extracts are cytotoxic and, thus, inhibit osteoclastogenesis. Therefore, it is important to investigate the cytotoxicity of FRB extracts. The cytotoxicity of FRB extract on the proliferation of osteoclast precursors was evaluated using Cell Counting Kit-8 (Dojin, Kumamoto, Japan) following the manufacturer’s recommendations. Osteoclast precursors were pre-cultured 1 × 10^4^ cells/well in 96-well plates with M-CSF (100 ng/mL) for 3 days. Thereafter, 0.5% DMSO or different concentrations of FRB extract (10, 50, 100, 500 μg/mL) were added to the newly changed medium. After 2 days, Cell Counting Kit-8 solution was added and incubated at 37 °C for 2 h. The cell viability was detected by evaluation of absorbance at 450 nm using a microplate reader (Remote Sunrise; Tecan, Kawasaki, Japan).

### 2.8. Primary Osteoblasts Isolation from Mice

Primary osteoblasts were obtained from the calvaria of neonatal mice. Neonatal mouse calvaria were incubated in 0.2% collagenase solution (3 mM K_2_HPO_4_, 10 mM NaHCO_3_, 70 mM NaCl, 25 mM HEPES, 1 mM CaCl_2_, 60 mM sorbitol, 0.5% glucose, 0.1% BSA) for 20 min or EDTA solution (PBS containing 5 mM EDTA 0.1% BSA; Wako, Japan) for 15 min on a shaker at 37 °C. Collagenase-treated digests were collected as Fraction 1 and EDTA-treated digests as Fraction 2. Additionally, then, the collagenase treatment was repeated two times to take fractions 3 and 4. These obtained cells from fractions 2–4 with high osteoblast content were collected and cultured in α-MEM. After 3 days of culture, adherent cells were considered osteoblasts and used in subsequent experiments.

### 2.9. Primary Peritoneal Macrophages Isolation from Mice

To obtain primary peritoneal macrophages, mice were intraperitoneally injected with sterile PBS (pH 7.4). After massaging the peritoneum, the liquid was aspirated and the cells were collected. The collected cells were cultured for 1 h. Floating cells were washed away, and after another 24 h of culture, adherent cells were considered macrophages and used in subsequent experiments.

### 2.10. Western Blotting Analysis

Osteoclast-associated cytokines act on osteoclast precursor cells to transduce various signaling during osteoclastogenesis, resulting in differentiation into osteoclasts. Therefore, it is important to investigate how FRB extract affects osteoclast-associated cytokines signaling in osteoclast precursors during osteoclastogenesis. We investigated how FRB extract affects transducing various signaling pathways during the inhibition of osteoclastogenesis. To exclude the effects of serum and M-CSF on signaling pathways, osteoclast precursors were starved in culture medium removing FBS and M-CSF for 6 h before adding reagents. Next, the cells were incubated with RANKL (100 ng/mL) or TNF-α (100 ng/mL) for specific times (0, 5, 15, 30, and 60 min) with or without FRB extract. The cells were lysed in RIPA lysis buffer, including protease and phosphatase inhibitor cocktail (Thermo Fisher Scientific, Rockford, IL, USA). After collecting the lysate, protein quantification was performed by using the BCA protein assay kit (Thermo Fisher Scientific). Lysates were treated by heating at 95 °C for 5 min. The lysates were then transferred to wells of 4–15% Mini-PROTEAN TGX Precast Gels (Bio-Rad, Hercules, CA, USA). Additionally, then, the lysates were electrophoresed. The separated protein bands were blotted onto PVDF membranes. The blotted membranes were incubated with Block-Ace (DS Pharma Biomedical, Osaka, Japan) to block nonspecific protein binding. After blocking, the rabbit monoclonal antibodies against phospho-ERK, phospho-JNK, phospho-p38, and phospho-IκB (Cell Signaling Technology, Danvers, MA, USA) were incubated at 1:1000 dilution overnight at 4 °C. Mouse monoclonal antibody against β-actin (Sigma-Aldrich) was also incubated at 1:1000 dilution overnight at 4 °C. After the reaction, the membranes were washed with TBS-T and TBS several times. Additionally, then, the membranes were incubated with HRP-conjugated anti-rabbit IgG antibody (Cell Signaling Technology) or anti-mouse antibody (GE Healthcare, Chicago, IL, USA) as secondary antibodies at 1:5000 dilution for 1 h at room temperature. Target proteins were visualized using SuperSignal West Femto Maximum Sensitivity Substrate (Thermo Fisher Scientific) and Fusion Fx chemiluminescence imaging system (Vilber Lourmat, Collégien, France). A quantitative analysis of band intensities was performed using image J software version 1.53a (NIH, Bethesda, MD, USA).

### 2.11. Statistical Analysis

The collected data are expressed as mean ± SD. Student’s *t*-test was used for comparison between the two groups, and Scheffé’s test was used for multiple comparisons. *p* < 0.05 was considered significant.

## 3. Results

### 3.1. FRB Supplementation Protected against LPS-Induced Inflammatory Osteoclastogenesis In Vivo

**Figure 1:**
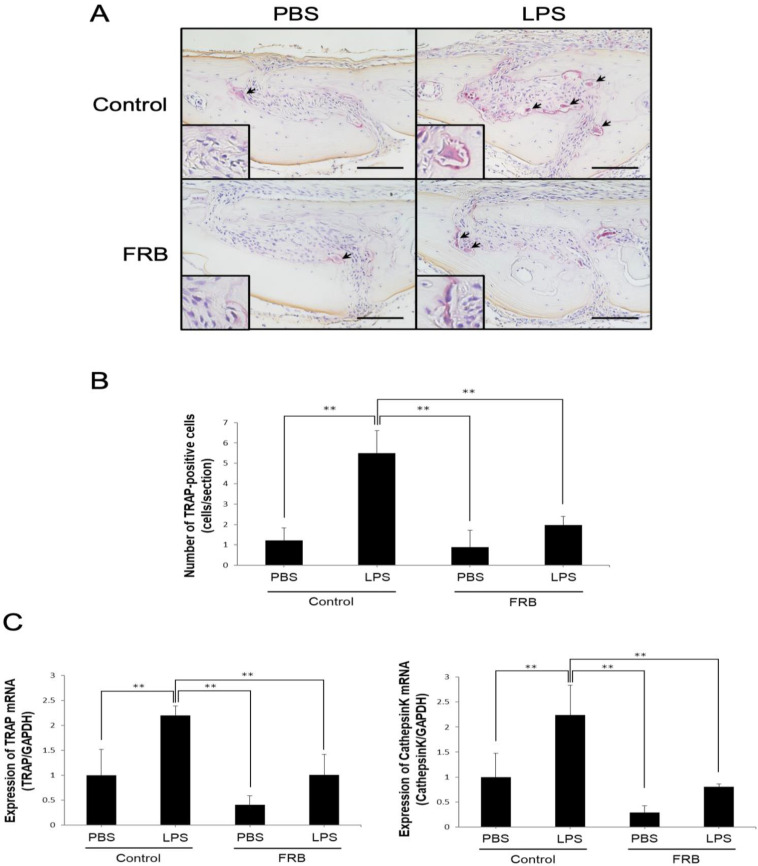
FRB supplementation inhibited osteoclast formation caused by LPS in mouse calvariae. (**A**) Representative images of TRAP stained calvarial sections from mice injected with PBS or LPS (100 µg/day) for 5 days under supplementation of control diet or FRB. Arrows indicate osteoclasts. (**B**) The number of TRAP-positive osteoclasts in the suture of mouse calvariae were analyzed. (**C**) TRAP and Cathepsin K mRNA expression levels in mouse calvaria were analyzed using real-time RT-PCR and were normalized relative to GAPDH. *n* = 4. Results are means ± SD. Significant differences among the groups are indicated as ** *p* < 0.01 using Scheffé’s test. Scale bars = 100 µm.

To evaluate the effect of FRB supplementation on LPS-induced osteoclast formation, we administered LPS into mouse calvariae with or without FRB supplementation. After 5 days of LPS administration, a large number of multinucleated TRAP-positive cells were observed in the suture mesenchyme, but the number was significantly lower in the FRB supplementation group than in the control diet supplementation group (Figure 1A,B). The real-time RT-PCR results also showed that in the control diet supplementation group, LPS administration increased the expression of osteoclast markers TRAP and cathepsin K more than PBS administration. In contrast, FRB supplementation decreased the expression of TRAP and cathepsin K, which was increased by LPS administration, compared to the control diet supplementation (Figure 1C).

### 3.2. FRB Supplementation Protected against LPS-Induced Bone Resorption In Vivo

**Figure 2:**
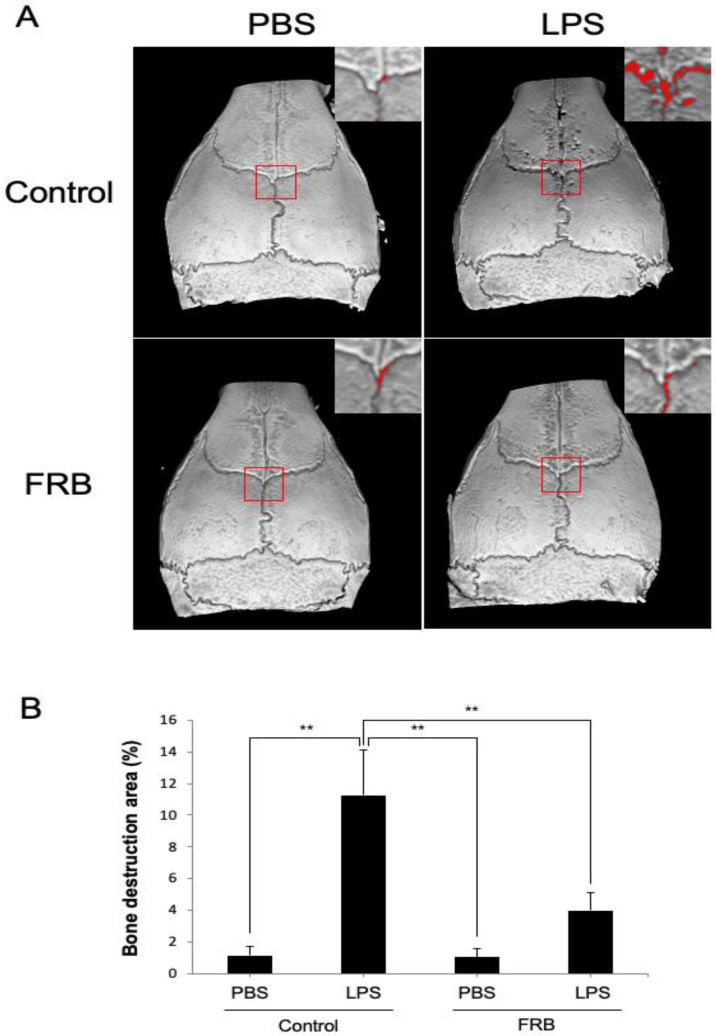
FRB supplementation inhibited born resorption caused by LPS in mouse calvariae. (**A**) Representative three-dimensional images of the calvariae from mice injected with PBS or LPS (100 µg/day) for 5 days under supplementation of control diet or FRB. Bone resorption areas are indicated by the red square border and the magnified image in the upper right corner in each image. (**B**) The percentage of bone resorption area to total calvarial bone area from mice in each treatment group. *n* = 4. Results are means ± SD. Significant differences among the groups are indicated as ** *p* < 0.01 using Scheffé’s test.

The calvaria of all mice were imaged with Micro-CT to measure the amount of bone resorption areas on the outer surface. In the control diet supplementation group, LPS administration significantly increased the ratio of bone resorption area to total area compared to PBS administration. In contrast, FRB supplementation reduced the increase in bone destruction caused by LPS administration (Figure 2A,B).

### 3.3. FRB Supplementation Downregulated the Expression of LPS-Induced Osteoclast-Associated Cytokines In Vivo

**Figure 3:**
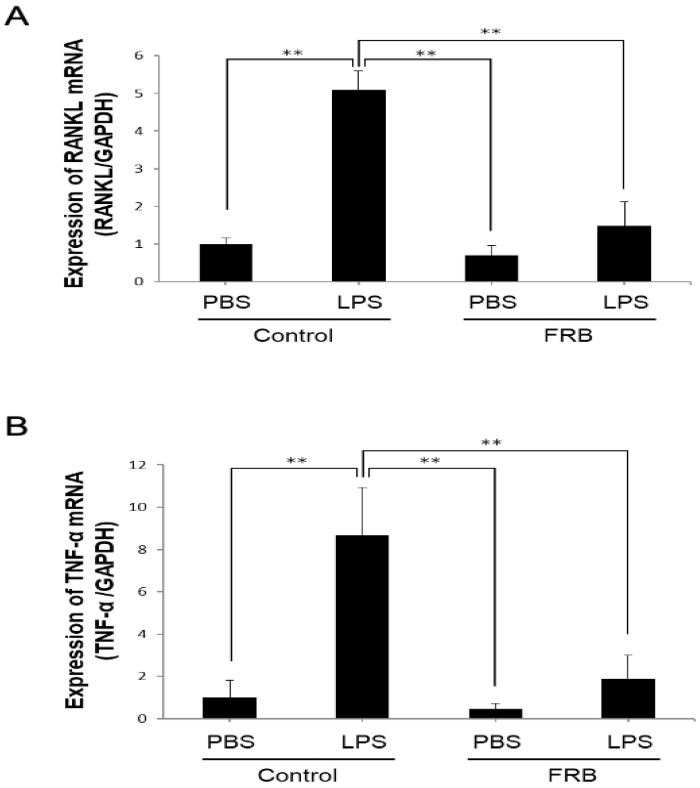
FRB supplementation inhibited expression of TNF-α and RANKL caused by LPS in mouse calvariae. (**A**) RANKL and (**B**) TNF-α mRNA expression levels in mouse calvaria were analyzed using real-time RT-PCR and were normalized relative to GAPDH. Total RNA was isolated from calvariae of mice injected with PBS or LPS (100 µg/day) for 5 days under supplementation of control diet or FRB. *n* = 4. Results are means ± SD. Significant differences among the groups are indicated as ** *p* < 0.01 using Scheffé’s test.

In vivo, the expression of the osteoclast-associated cytokines RANKL and TNF-α plays an important role in LPS-induced inflammatory osteoclast differentiation. Therefore, to examine the effect of FRB on the expression of LPS-induced osteoclast-associated cytokines induced in mouse calvaria, RANKL and TNF-α mRNA expression was measured using real-time RT-PCR. mRNA expression of RANKL was increased by the LPS treatment compared to the PBS treatment. However, LPS-induced RANKL mRNA expression was reduced by FRB supplementation (Figure 3A). Similar to RANKL, the expression of TNF-α, which was also elevated by LPS, was suppressed by FRB supplementation (Figure 3B).

### 3.4. FRB Inhibited Osteoclast Formation Caused by RANKL and TNF-α but Not Cell Viability of Osteoclast Precursor Cells

**Figure 4:**
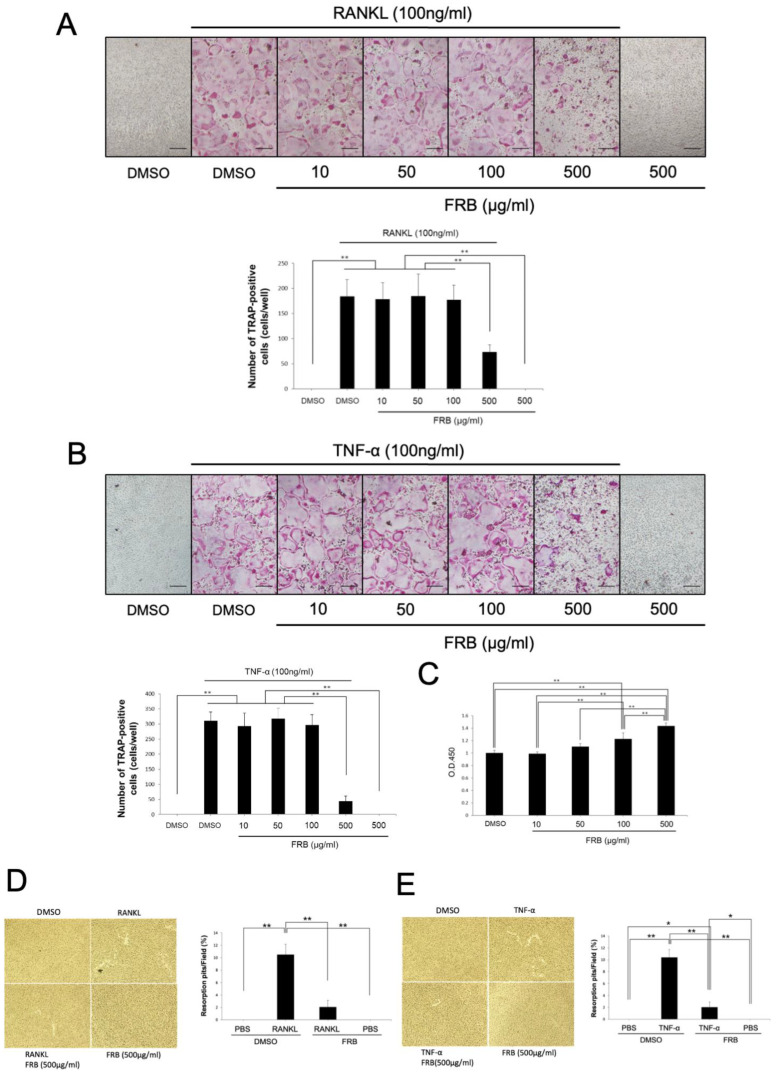
FRB extract inhibited osteoclast formation caused by RANKL or TNF-α but not cell viability of osteoclast precursor cells. (**A**) Representative images of TRAP staining of osteoclast precursors treated with M-CSF alone or M-CSF and RANKL in the presence of 0.5% DMSO (control vehicle) or the indicated concentrations of FRB extract for 5 days. The number of TRAP-positive osteoclasts in each well were analyzed. (**B**) Representative images of TRAP staining of osteoclast precursors treated with M-CSF alone or M-CSF and TNF-α in the presence of 0.5% DMSO (control vehicle) or the indicated concentrations of FRB extract for 5 days. The number of TRAP-positive osteoclasts in each well was analyzed. (**C**) Osteoclast precursors were incubated with different concentrations of FRB extract in the presence of M-CSF. After 2 days, cell viability was evaluated using CCK-8 assay. (**D**) Microscopic images of RANKL generated osteoclast-induced bone resorption and percentage of resorption pits. (**E**) Microscopic images of TNF-a generated osteoclast-induced bone resorption and percentage of resorption pits. *n* = 4. Results are means ± SD. Significant differences among the groups are indicated as * *p* < 0.05, ** *p* < 0.01 using Scheffé’s test. Scale bars = 200 µm.

To reveal the mechanism of inhibition of osteoclastogenesis by FRB in vivo, we investigated whether FRB directly affects RANKL- and TNF-α-induced osteoclastogenesis in vitro by using TRAP staining. We also examined whether the effect of concentration on osteoclastogenesis varied. TRAP-positive cells were induced by adding RANKL or TNF-α along with M-CSF to osteoclast precursor cultures. The results showed no difference in the number of TRAP-positive cells at concentrations up to 100 μg/mL when FRB extract was applied simultaneously to these cultures. However, FRB extract significantly reduced the number of TRAP-positive cells at a concentration of 500 μg/mL (Figure 4A,B). In addition, we investigated whether the attenuating effect of FRB extract on osteoclastogenesis is related to cell viability of osteoclast precursors. It is possible that FRB extracts are cytotoxic and thus inhibit osteoclastogenesis. Therefore, it is important to investigate the cytotoxicity of FRB extracts. Rather than decreasing the cell viability of osteoclast precursors in cultures treated with M-CSF and FRB extract, the proliferation of osteoclast precursors increased in an FRB concentration-dependent manner (Figure 4C). We checked whether the FRB extract was absorbed at 459 nm. All the FRB extract doses did not absorb at 45 m. Furthermore, FRB extract (500 μg/mL) inhibited both resorption pit induced by RANKL (Figure 4D) and TNF-α (Figure 4E). These results suggest that the osteoclast-inhibitory effect of FRB in vivo is due to the direct action of FRB on osteoclast precursors and not to its cytotoxic effects. Because of the significant effect on osteoclastogenesis, the concentration of FRB used in subsequent in vitro experiments was determined at 500 μg/mL.

### 3.5. FRB Inhibited the Phosphorylation of IκB Caused by RANKL and TNF-α in Osteoclast Precursor Cells

**Figure 5:**
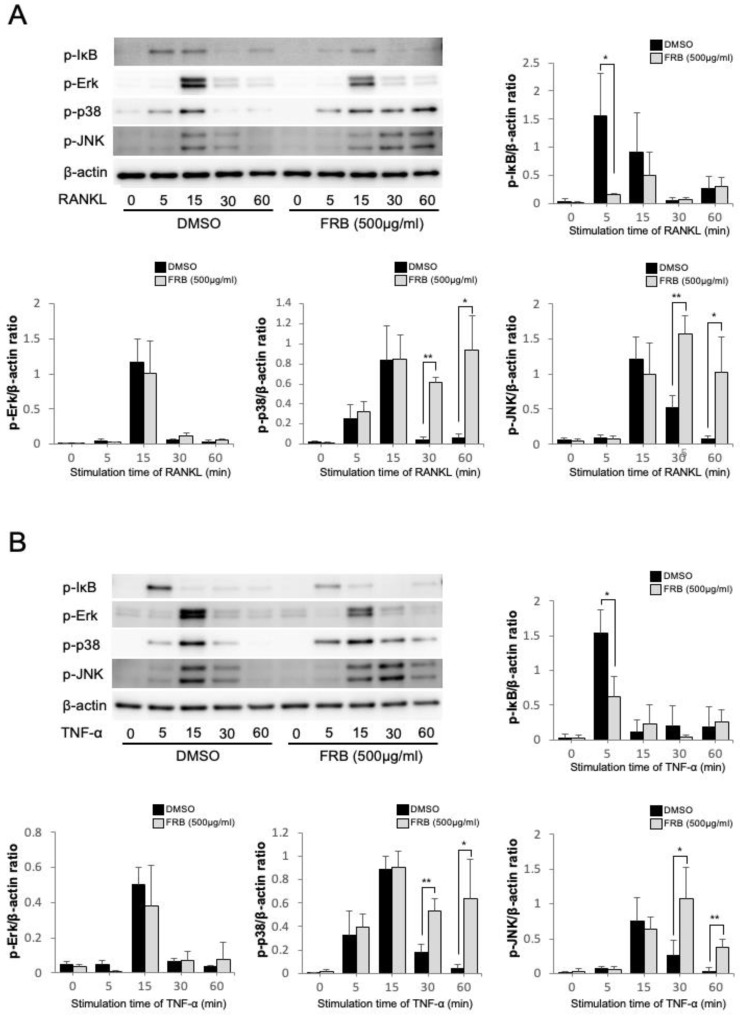
Effect of FRB extract on IκB and MAPKs (Erk, P38, and JNK) phosphorylation in RANKL or TNF-α induced osteoclast precursors. (**A**) Osteoclast precursors were treated with RANKL in the presence of 0.5% DMSO (control vehicle) or FRB extract (500 µg/mL) for the indicated times. Phosphorylated IκB and MAPKs (Erk, JNK, and P38) were detected with Western blotting. The band intensity ratios of phosphorylated IκB and MAPKs (Erk, JNK, and P38) to β-actin were quantified, respectively. (**B**) Osteoclast precursors were treated with TNF-α in the presence of 0.5% DMSO (control vehicle) or FRB extract (500 µg/mL) for the indicated times. After the cells were lysed, Western blot analysis was performed using specific antibodies. The band intensity ratios of phosphorylated IκB and MAPKs (Erk, JNK, and P38) to β-actin were quantified, respectively. *n* = 3. Results are means ± SD. Significant differences between the two groups (control group and FRB extract treated group) are indicated as * *p* < 0.05, ** *p* < 0.01 using Student’s *t*-test.

Osteoclast-associated cytokines act on osteoclast precursor cells to transduce various signaling during osteoclastogenesis, resulting in differentiation into osteoclasts. Thus, it is important to investigate how FRB extract affects osteoclast-associated cytokines signaling in osteoclast precursors during osteoclastogenesis. Therefore, we investigated how FRB extract affects the transduction of various signaling pathways during the inhibition of osteoclastogenesis. The NF-κB and MAPK pathways activated by RANKL and TNF-α play an important role in osteoclastogenesis. Thus, we investigated which signaling pathways were affected by the FRB extract. The phosphorylation of MAPKs (Erk, p38, and JNK) and IκB was detected by specific antibodies. Stimulation of osteoclast precursors with RANKL or TNF-α increased phosphorylation of IκB after 5 min, but phosphorylation of IκB was inhibited when FRB was applied together (Figure 5A,B). The stimulation of osteoclast precursors with RANKL or TNF-α also increased the phosphorylation of Erk, p38, and JNK at 15 min. There was no significant difference in these phosphorylations when FRB was applied together. On the other hand, FRB enhanced the phosphorylation of p38 and JNK at 30 min and 60 min (Figure 5A,B). There was no significant effect on the phosphorylation of Erk by FRB (Figure 5A,B). We confirmed these results through a comparison of total protein and phosphorylated protein induced by RANKL and TNF-α. The results also showed that FRB extract inhibited the phosphorylation of IκB caused by RANKL and TNF-α in osteoclast precursor cells (Figure S1). The NF-κB pathway may be involved in the inhibitory effect of FRB on osteoclastogenesis. 

### 3.6. FRB Did Not Affect LPS-Induced RANKL Expression in Osteoblasts

**Figure 6:**
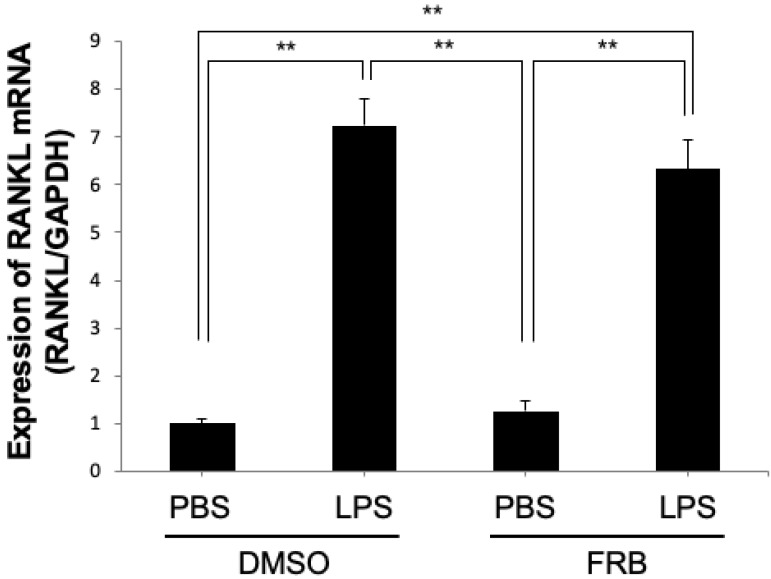
FRB extract had no effect on expression of RANKL caused by LPS in osteoblasts. RANKL mRNA expression levels in osteoblasts treated with PBS or LPS for 3 days in the presence of 0.5% DMSO (control vehicle) or FRB extract (500 μg/mL) were analyzed using real-time RT-PCR. GAPDH was used for normalization. *n* = 4. Results are means ± SD. Significant differences among the groups are indicated as ** *p* < 0.01 using Scheffé’s test.

Osteoblasts have been shown to be involved in osteoclastogenesis through increased RANKL expression upon LPS stimulation [43,44]. We analyzed the mRNA expression levels of RANKL in osteoblasts induced by LPS to determine the mechanism of the inhibitory effect of FRB in vitro. The mRNA expression level of RANKL was increased in both groups treated with LPS with or without FRB extract. However, there was no significant difference in RANKL expression between the FRB extract treatment or non-treatment osteoblasts (Figure 6). FRB may not be involved in RANKL expression in LPS-stimulated osteoblasts.

### 3.7. FRB Suppressed LPS-Induced TNF-α Expression in Macrophages

**Figure 7:**
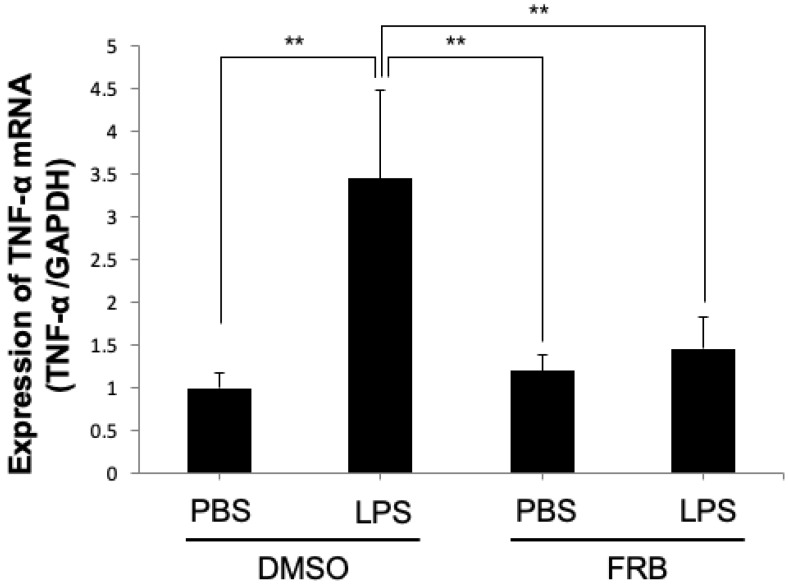
FRB extract inhibited the expression of TNF-α caused by LPS in macrophages. TNF-α mRNA expression levels in macrophages treated with PBS or LPS for 3 days in the presence of 0.5% DMSO (control vehicle) or FRB extract (500 μg/mL) were analyzed using real-time RT-PCR. GAPDH was used for normalization. *n* = 4. Results are means ± SD. Significant differences among the groups are indicated as ** *p* < 0.01 using Scheffé’s test.

Macrophages, one of the immune cells, are also factors that promote osteoclast differentiation by producing TNF-α upon LPS stimulation [43,44]. To further investigate how FRB inhibits LPS-induced osteoclastogenesis in vivo, we examined whether FRB inhibits LPS-induced TNF-α expression in macrophages in vitro. The real-time RT-PCR results showed that TNF-α mRNA expression was increased by LPS treatment, but LPS-induced TNF-α expression was decreased by FRB extract treatment (Figure 7). FRB may have an inhibitory effect on TNF-α expression in LPS-stimulated macrophages.

## 4. Discussion

Diseases of pathological bone resorption accompanied by inflammation, such as aseptic loosening, periodontal disease, and rheumatoid arthritis, have a profound negative impact on daily activities and significantly diminish the overall quality of life. Therefore, it is crucial to understand the underlying mechanisms of these diseases and explore methods to manage their detrimental impact. Several reports showed that FRB has anti-inflammatory effects. Therefore, we hypothesized that FRB could suppress diseases associated with pathological bone resorption caused by inflammation. LPS is known as a major causal factor of osteolytic diseases induced by inflammation. Therefore, finding ways to inhibit pathological bone resorption caused by LPS-induced inflammation can reduce the disadvantages induced by osteolytic diseases induced by inflammation such as periodontitis, septic arthritis, osteomyelitis, and implant infections. In this study, we produced FRB via double fermentation with *Aspergillus kawachii* and three kinds of lactic acid bacteria. Then, we evaluated how supplementation with double-fermented FBR affected osteoclastogenesis and bone resorption. The results showed that inflammation-induced osteoclastogenesis and bone resorption were suppressed in FRB-supplemented mice compared to control diet-supplemented mice. Since LPS increases the osteoclast-associated cytokines RANKL and TNF-α when administered to mice, we examined how FRB supplementation affects LPS-induced RANKL and TNF-α production. As a result, FRB supplementation inhibited LPS-induced RANKL and TNF-α production compared to control-diet supplemented mice. Based on these findings, we investigated whether FRB extract affects osteoclastogenesis induced by RANKL and TNF-α. The results showed that FRB extract directly inhibited osteoclast formation induced by RANKL and TNF-α. Since LPS acts on macrophages to increase TNF-α expression, we examined whether FRB extract acts on macrophages to suppress the increase in TNF-α expression. FRB extract directly inhibited LPS-induced TNF-α expression in macrophages in vitro. LPS is known to act on osteoblasts and increase the expression of RANKL. Therefore, we examined whether FRB extract acts on osteoblasts and suppresses the increase in RANKL expression. However, FRB extract cannot suppress RANKL expression induced by LPS in osteoblasts. The present study is an initial report which describes the effects and mechanisms of FRB supplementation on LPS-induced osteoclastogenesis and bone resorption.

A byproduct of the rice milling industry, RB has been known to be highly nutritious. It is believed that fermentation alters the bioavailability and composition of bioactive compounds in RB. However, RB is usually discarded or used as livestock feed. In this research, a product digested by *Aspergillus kawachii* was further fermented by three lactic acid bacteria. This combination of microorganisms was hoped to promote the nutritional value of RB. In former reports, FRB has been shown to protect the intestines of mice from DDS-induced colitis [33] and to be capable of reducing inflammatory molecules in the muscles atrophy of diabetes rat model [36]. This special process of fermentation has been shown to increase the nutritive value of RB, especially its total phenolic content [37]. Several studies showed that fermented rice and RB without fermentation inhibited osteoclast formation. Black rice fermented with *Lactobacillus casei* inhibits osteoclast formation and osteoporosis induced by ovariectomy [45]. Brown rice extract treated with *Lactobacillus sakei* Wikim001 inhibits osteoclast formation, but enhances osteoblast activities [46]. However, there is no finding of the effect of FRB supplementation on osteoclast formation and bone resorption induced by LPS. The fermented RB supplementation attenuated osteoclastogenesis induced by LPS as inflammation in vivo compared to control diet supplementation. We also investigated the suppressing effect of FRB supplementation on bone resorption. The severity of bone resorption was evaluated by the micro-CT images of each group. The results showed that the degree of bone resorption was much lower in the FRB supplementation and LPS-administrated mice than in the control diet and LPS-administrated mice. These results indicated that FRB supplementation inhibits osteoclast formation and bone resorption induced by LPS in vivo. In other words, our findings regarded the suppressive effect of FRB supplementation on inflammation-induced osteoclastogenesis and bone resorption.

Based on these results, we discuss the mechanism by which FRB supplementation suppresses osteoclast formation and bone resorption in vivo. We thought of two mechanisms for the proposed suppressive effect of FRB on osteoclast formation and bone resorption induced by LPS in vivo. One possibility is that FRB suppressed the expression of LPS-induced inflammatory cytokines associated with osteoclastogenesis, such as RANKL and TNF-α. Osteoclast formation would be inhibited when these cytokines are both downregulated. A number of studies have shown that LPS leads to in vivo increasing expression of osteoclast-associated cytokines such as TNF-α and RANKL [15,47]. Furthermore, it is widely recognized that RANKL is a crucial factor for formation [5] and that also TNF-α is reported to induce osteoclast formation in mice [8,9]. In the present study, both mRNA levels of RANKL and TNF-α were decreased in mice injected LPS with FRB supplementation compared with mice administered LPS with control diet supplementation. These results indicated that one of the mechanisms underlining the suppressive effect of FRB supplementation on LPS-induced osteoclast formation might be the reduction in osteoclast-related cytokines RANKL and TNF-α induced by LPS. It has been reported that this FRB supplementation inhibits TNF-α expression in DSS-induced inflammation [35] in chronic colitis-associated extraintestinal manifestations [34] and in streptozotocin-induced diabetic rats [36]. It may be that FRB supplementation systemically inhibits TNF-α expression in vivo. A second mechanism might involve that FRB directly suppressed both RANKL- and TNF-α-induced osteoclast formation. Thus, we examined that FRB exerts its inhibitory effect on osteoclasts by acting directly on osteoclast progenitor cells. The results indicated that FRB extract directly inhibited both RANKL-induced and TNF-α-induced differentiation of osteoclast progenitor cells into osteoclasts. Moreover, we investigated whether FRB extract could inhibit the viability of osteoclast precursors because it is possible that FRB extracts are cytotoxic and thus inhibit osteoclastogenesis. Therefore, it was important to investigate the cytotoxicity of FRB extracts. However, FRB extract did not inhibit osteoclast precursor viability but increased cell proliferation of osteoclast precursors. According to these results, the suppressive effects of FRB extract on osteoclast formation are due to both decreasing osteoclast-related cytokines and the direct inhibition of osteoclast precursors in vivo.

During osteoclastogenesis, osteoclast-associated cytokines act on osteoclast precursor cells to transduce various signaling, resulting in differentiation into osteoclasts. Therefore, it is important to investigate how FRB extract affects osteoclast-associated cytokines signaling in osteoclast precursors during osteoclastogenesis. It has been reported that RANKL promotes the phosphorylation of MAPKs, such as JNK, p38 and ERK, and IκB in osteoclast precursors [48]. Therefore, we investigated how FRB extract affects the transduction of various signals during osteoclast formation inhibition. In the present study, we assessed the effect of FRB extract on the RANKL-induced phosphorylation of MAPKs (JNK, p38, and Erk) and IκB by specific antibodies. The results showed that FRB extract inhibited the phosphorylation of IκB when cells were treated with RANKL. However, FRB extract did not inhibit the phosphorylation of JNK, p38 and ERK. The results suggested that suppression of the RANKL-induced phosphorylation of IκB by FRB extract was thought to play an important role in the inhibitory effect of FRB extract on RANKL-induced osteoclast formation. Previously, we described how TNF-α promotes the phosphorylation of MAPKs, including JNK, p38 and ERK, in osteoclast progenitors [14]. In addition, we have also reported that, in osteoclast precursors, TNF-α induces the phosphorylation of IκB [41]. In the present study, we evaluated the effect of FRB extract for the TNF-α-induced phosphorylation of MAPKs and IκB. FRB extract also inhibited the phosphorylation of IκB when cells were treated with TNF-α. However, FRB extract did not inhibit the phosphorylation of JNK, p38 and ERK. The results suggest that the suppression of the TNF-α-induced phosphorylation of IκB by FRB extract also plays a major role in the inhibitory effect of FRB extract on RANKL-induced osteoclast formation.

Next, we confirmed that FRB extract suppresses TNF-α mRNA expression in macrophages induced by LPS, indicating that the downregulation of LPS-induced TNF-α expression by FRB extract may result from a direct inhibition of FRB extract on macrophages. We also confirmed whether FRB extract inhibited LPS-induced RANKL mRNA expression in osteoblasts, which were obtained from calvariae in neonatal mice. However, there was no effect of FRB extract on the LPS-induced RANKL expression of osteoblasts. The results suggested that there is no direct action of FRB extract on osteoblasts. In this study, we found that FRB supplementation inhibited inflammation-induced osteoclast formation and bone resorption. Rice bran is usually discarded or used as livestock feed, but it was found that fermenting rice bran increases the active ingredients and promotes health for humans. It has been found that fermenting rice bran can be used effectively. Osteoporosis is one of the important osteolytic diseases caused by osteoclastic bone resorption. In the future, it will be necessary to see whether FRB supplementation works to prevent bone resorption associated with osteoporosis. We also found that the oral administration of FRB inhibited local osteoclastogenesis, which means FRB supplementation had a systemic effect. Oral administration is the most convenient and usually the safest and least expensive administration method. Another advantage is that it is easy to prepare with long-lasting action, such as an extended-release tablet. For this reason, the FRB may also have the advantage of being easy to use as an orally administered medicine or supplement. In the future, it is necessary to investigate in detail which components of FRB work to suppress osteoclastogenesis, and to conduct research to more effectively treat pathological osteoclastogenesis using FRB.

It has been reported that feeding FRBs increased the amount of short-chain fatty acids in the intestine and suppressed enteritis in a colitis model via GRP43 [33]. It has also been reported that tryptophan, which is relatively abundant in FRB, is metabolized by intestinal bacteria to tryptamine and other indole compounds, which act as ligands for aryl hydrocarbon receptors (AHRs) and exhibit anti-inflammatory effects [38,49]. Thus, there is a possibility that FRB ingestion may have a postbiotic effect, in which metabolites by intestinal bacteria have a beneficial effect on the host. The effect of FRBs on osteoclastogenesis and bone resorption reported in this study may include a postbiotic effect. This point should be studied in the future. In vitro experiments also cannot determine whether metabolites of FRB produced by intestinal bacteria have an inhibitory effect on osteoclastogenesis. Further experiments are needed in the future.

## 5. Conclusions

**Figure 8:**
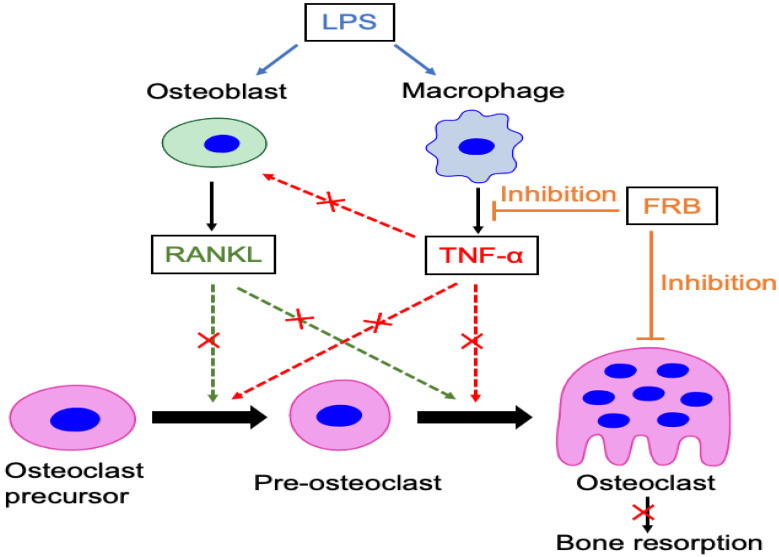
Schematic of the inhibitory mechanism of FRB supplementation on LPS-induced osteoclastogenesis and bone resorption. These effects may be involved in the suppression of osteoclast formation caused by the osteoclast-related cytokines RANKL and TNF-α and the suppression of TNF-α production in macrophages caused by LPS. Furthermore, although FRB has no direct effect on RANKL expression in osteoblasts during LPS stimulation, it may indirectly suppress RANKL expression in osteoblasts by inhibiting TNF-α production by macrophages. FRB directly and indirectly may inhibit osteoclast formation and bone resorption induced by LPS in vivo.

Osteolytic disorders cause pathological bone resorption induced by osteoclasts with inflammation, resulting in a reduction in quality of life. Therefore, it is a significant mission to clarify the mechanisms of these diseases and to find ways to treat them. In this study, our experimental results show that supplementation with FRB—which was made using two-step fermentation—suppressed osteoclast formation and bone resorption induced by LPS administration in the calvariae of mice. Furthermore, FRB extract directly inhibited osteoclast formation induced by both TNF-α and RANKL in mice and in osteoclast precursor cells in vitro. The possible mechanisms for the suppressive effect of FRB intake on osteoclast formation and bone resorption induced by LPS in vivo might be related to the suppression of LPS-induced TNF-α production in macrophages and the direct inhibition of osteoclast formation induced by TNF-α and RANKL (Figure 8). Rice bran is usually discarded or used as feed for livestock, but it was found that fermenting rice bran increases the active ingredients and promotes health for humans. We also found that the oral administration of FRB inhibited local osteoclastogenesis. FRB may also have the advantage of being easy to use as an orally administered medicine or supplement. The findings of the present study suggested that FRB has the potential as a therapeutic regent for anti-osteolytic conditions. Finally, there is a possibility of a postbiotic effect, in which metabolites by intestinal bacteria have a beneficial effect on the host due to FRB intake, and that further research is needed.

## Data Availability

The data presented in this study are available on request from the corresponding author.

## Supplementary Materials

The following supporting information can be downloaded at: https://www.mdpi.com/article/10.3390/nu15133044/s1, Figure S1: Comparison of total form protein and phosphorylated protein induced by RANKL and TNF-α.
