# Supplementary material for: Fermented Rice Bran Supplementation Inhibits LPS-Induced Osteoclast Formation and Bone Resorption in Mice
Source: Nutrients. 2023 Jul 5;15(13):3044. doi: 10.3390/nu15133044 (PMC10346360; doi:10.3390/nu15133044)
Supplement: Supplementary file 1 [file nutrients-15-03044-s001.zip › nutrients-2454163-supplementary.pdf]

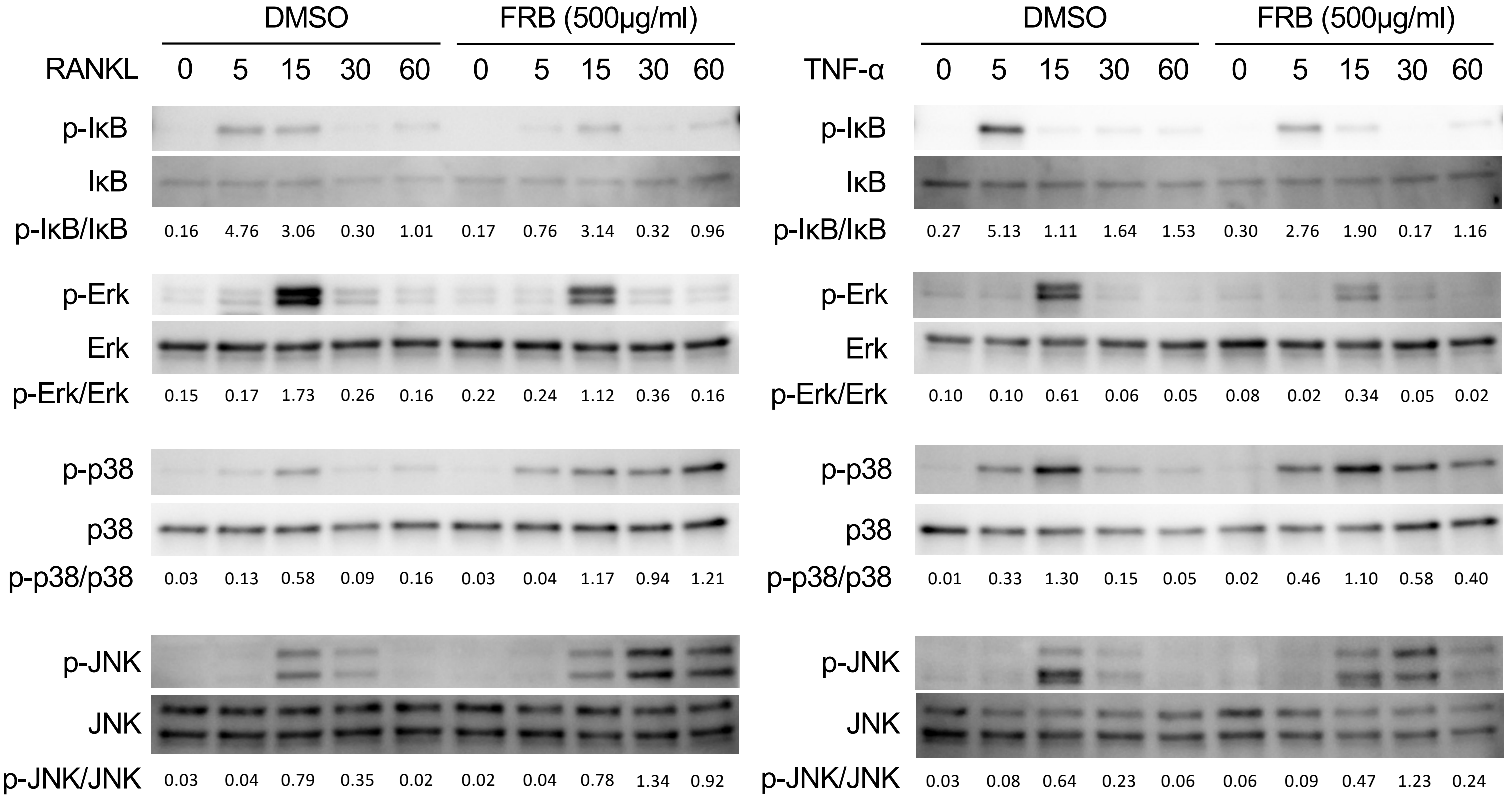

Figure S1. Comparison of total form protein and phosphorylated protein induced by RANKL and TNF-α. To exclude the effects of serum and M-CSF on signaling pathways, osteoclast precursors were starved in culture medium removing FBS and M-CSF for 6 hours before adding reagents. Next, the cells were incubated with RANKL (100 ng/ml) or TNF-α (100 ng/ml) for specific times (0, 5, 15, 30, 60 minutes) with or without FRB extract. The cells were lysed in RIPA lysis buffer including protease and phosphatase inhibitor cocktail (Thermo Fisher Scientific, Rockford, IL, USA). After collecting lysate, protein quantification was per-formed by using the BCA protein assay kit (Thermo Fisher Scientific). Lysates were treated by heating at 95° C for 5 min. The lysates were then transferred to wells of 4% - 15% Mini-PROTEAN TGX Precast Gels (Bio-Rad). And then the lysates electrophoresed. The separated protein bands were blotted onto PVDF membranes. The blotted membranes were incubated with Block-Ace (DS Pharma Biomedical, Osaka, Japan) to block non-specific protein binding. After blocking, the rabbit monoclonal antibodies against ERK, JNK, p38, and IκB (Cell Signaling Technology, Danvers, MA, USA) and phospho-ERK, phospho-JNK, phospho-p38, and phospho-IκB (Cell Signaling Technology, Danvers, MA, USA) were incubated at 1:1000 dilution overnight at 4° C. After reaction, the membranes were washed with TBS-T and TBS several times. And then, the membranes were incubated with HRP-conjugated anti-rabbit IgG antibody (Cell Signaling Technology) as secondary antibodies at 1:5000 dilution for 1 hour at room temperature. Target proteins were visualized using SuperSignal West Femto Maximum Sensitivity Substrate (Thermo Fisher Scientific) and Fusion Fx chemiluminescence imaging system (Vilber Lourmat, Collégien, France). Quantitative analysis of band intensities was performed using image J software (NIH). The results showed that FRB extract inhibited phosphorylation of IκB caused by RANKL and TNF-α in osteoclast precursor cells.
